# Observational study of the heterogeneous global meteotsunami generated after the Hunga Tonga–Hunga Ha’apai Volcano eruption

**DOI:** 10.1038/s41598-023-35800-6

**Published:** 2023-05-27

**Authors:** Joan Villalonga, Àngel Amores, Sebastià Monserrat, Marta Marcos, Damià Gomis, Gabriel Jordà

**Affiliations:** 1 Centre Oceanogràfic de Balears, CN-Instituto Español de Oceanografía (IEO-CSIC), Palma, Spain; 2 Departament de Física, Universitat de Les Illes Balears (UIB), Palma, Spain; 3 Institut Mediterrani d’Estudis Avançats (UIB-CSIC), Esporles, Spain

**Keywords:** Natural hazards, Physical oceanography, Atmospheric dynamics

## Abstract

The Hunga Tonga–Hunga Ha’apai volcano eruption of January 15th 2022 generated a global atmospheric and oceanic response that was recorded by an unprecedented amount of sensors. The eruption caused an atmospheric perturbation that travelled as a Lamb wave surrounding the Earth at least 3 times, and was recorded by hundreds of barographs worldwide. The atmospheric wave showed complex patterns of amplitude and spectral energy content, although most of the energy was concentrated in the band (2–120 min). Simultaneously to each passage of the atmospheric wave and after, significant Sea Level Oscillations (SLOs) in the tsunami frequency band were recorded by tide gauges located all around the globe, in what it can be referred to as a global meteotsunami. The amplitude and dominant frequency of the recorded SLOs showed a high spatial heterogeneity. Our point is that the geometry of continental shelves and harbours acted as tuners for the surface waves generated by the atmospheric disturbance at open sea, amplifying the signal at the eigenmodes of each shelf and harbour.

## Introduction

Meteotsunamis are high frequency Sea Level Oscillations (SLOs) with spectral energy in the same frequency band than tsunamis (2–120 min). The difference with those is that the triggering processes are atmospheric disturbances (e.g., atmospheric gravity waves, convective systems…)^1–3^. The direct sea level response to changes in atmospheric pressure is approximately of 1 cm for each hPa of change in the pressure exerted by the air column above (usually referred to as the inverted barometer response). For the meteotsunamis to reach hazardous oscillation amplitudes at the coast, some physical mechanism (e.g., Proudman resonance, harbour resonance…) is required to amplify the sea level response, since changes in atmospheric pressure are typically of a few mbar. Meteotsunamis have been reported worldwide, but always as a local or regional process^4^, since the forcing atmospheric disturbances rarely affect more than one region. In fact, the occurrence of a meteotsunami with a global dimension is such an extraordinary event that it has only been reported twice in the era of modern scientific instrumentation: after the Krakatoa volcano eruption, in 1883, and after the recent eruption of the Hunga Tonga–Hunga Ha’apai volcano, in January 2022.

The explosion of the Krakatoa volcano, located in the Sunda Strait, generated atmospheric and oceanic signals at the global scale. The atmospheric disturbance associated with that eruption was detected in barograph records, circling the Earth up to 3 times^5^. SLOs were also reported worldwide, caused either by the tsunami generated by the explosion at the volcano site^6^, or by the atmospheric wave triggered by the explosion^7–9^. While the first were restricted to the Indonesian Archipelago, the second were detected in regions far from the source, with timings coinciding with the passage of the atmospheric pressure disturbance^7,9^. Thus, the Krakatoa event can be considered as the first reported global meteotsunami, though the number of observations was relatively small at that time. It must also be noticed that the term ‘meteotsunami’ became widely accepted much later, after the publication of Ref.^1^ in 2006.

The eruption on January 15th 2022 of the Hunga Tonga–Hunga Ha’apai volcano, located in the South Pacific Tonga archipelago, was also an extraordinary event that liberated an energy equivalent to the explosion of 59–63 megatons of TNT^10^. The eruption and its impacts were recorded by an unprecedented amount of observations (e.g., the satellite images of the explosion became viral worldwide; see Supplementary Information and Ref.^11^). The most affected region was the Tonga archipelago, which was covered by the volcano ash and where the tsunami wave resulting from the explosion caused four casualties and damages estimated in $90.4 million dollars^12^.

The explosion generated noticeable perturbations in both the atmosphere and the ocean, which propagated far from the source and were felt in remote regions of the globe up to several days after the explosion. The atmospheric disturbance was modeled at the global scale by Ref.^13^, who showed that it propagated as a Lamb-type pressure wave travelling with a speed close to the speed of sound. In the ocean, the tsunami waves generated by the collapse of the volcano propagated all over the Pacific Ocean and were recorded by the DART system (Deep-ocean Assessment and Reporting of Tsunamis**,** https://www.ngdc.noaa.gov/hazel/view/hazards/tsunami/related-runups/5824); the amplitude of the recorded SLOs ranged from a few centimeters up to 1–2 m at some tide gauges of Japan and the West Coast of the USA, Mexico, Chile and Peru^14^. Studies combining the analysis of observations with numerical simulations have identified that, in the Pacific Ocean, the observed SLOs could be attributed to three different processes: (i) the long (tsunami) waves generated by the collapse of the volcano; (ii) the sea level response to an atmospheric shock wave in the vicinity of the volcano; and (iii) the sea level response to the atmospheric Lamb waves that propagated far from the source^15–20^. The overlapping of the three processes made that in the Pacific Ocean SLOs were significantly larger than in the rest of the globe.

Away from the Pacific Ocean, SLOs would be entirely related to the propagation of the atmospheric Lamb waves. Given the global dimension of these waves and of the resulting meteotsunami, one could expect some worldwide homogeneity in the characteristics of the sea level response. However, the fact is that there are marked differences between tide gauges records, even between those located in the same region. In a first analysis, Ref.^19^ tried to relate the timings and amplitudes recorded by tide gauges located worldwide with the different generating processes outlined above; however, they did not examine in detail the causes of the observed spatial heterogeneity. Other works have performed global numerical simulations of the atmosphere–ocean coupling that resulted in the ocean long waves observed outside the Pacific Ocean^21,22^; however, these studies cannot reproduce the sea level oscillations observed by coastal tide gauges, because this would require high resolution simulations with very high-resolution bathymetries near the coast (which is prohibitive for global-scale simulations).

Here we try to explain the spatial heterogeneity of the observed sea level response at the coast through an exhaustive analysis of atmospheric pressure and sea level observations recorded worldwide. On one hand, we intend to give an overall description of the observations to highlight the global dimension of both, the atmospheric pressure disturbance and the sea level response. On the other hand, we focus on the amplitudes and spectral contents of time series to gain some insight in the causes of the observed spatial heterogeneity. We do this first for the atmospheric pressure time series (next section) and later for the sea level time series (section after the next). In the last section results are discussed altogether in order to reach some conclusions.

### The atmospheric perturbation triggered by the volcano explosion

**Figure 1:**
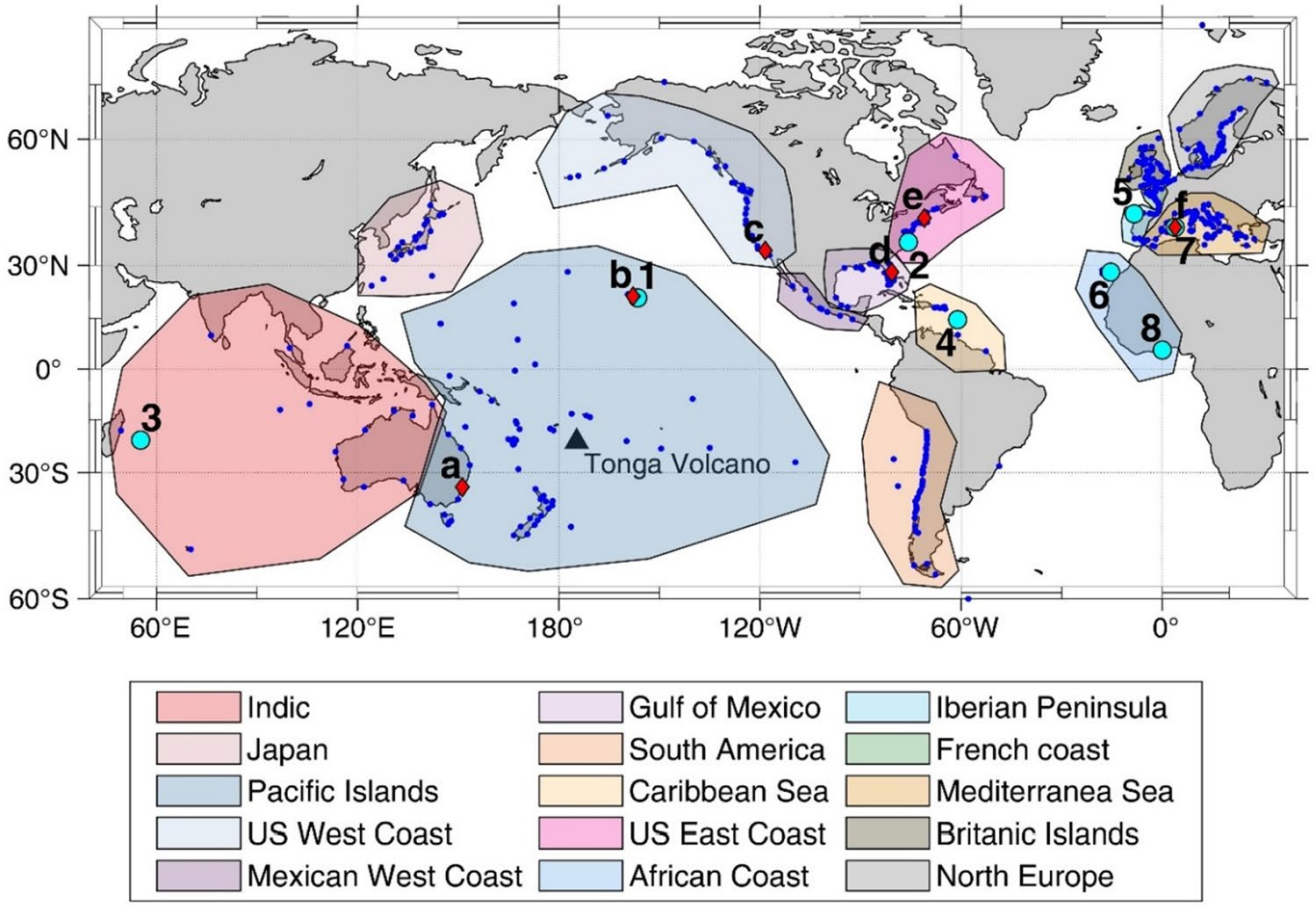
Location of the tide gauges used in this work (small blue dots). The red diamonds with letters show the location of the atmospheric pressure time series plotted in Fig. 2. The cyan dots with numbers show the location of the sea level time series plotted in Fig. 4. The colored patches show the different world regions selected to perform the analysis shown in Fig. 5. The map has been drawn with MATLAB^33^ using the M_Map^34^ toolbox (https://www.eoas.ubc.ca/~rich/map.html).

**Figure 2:**
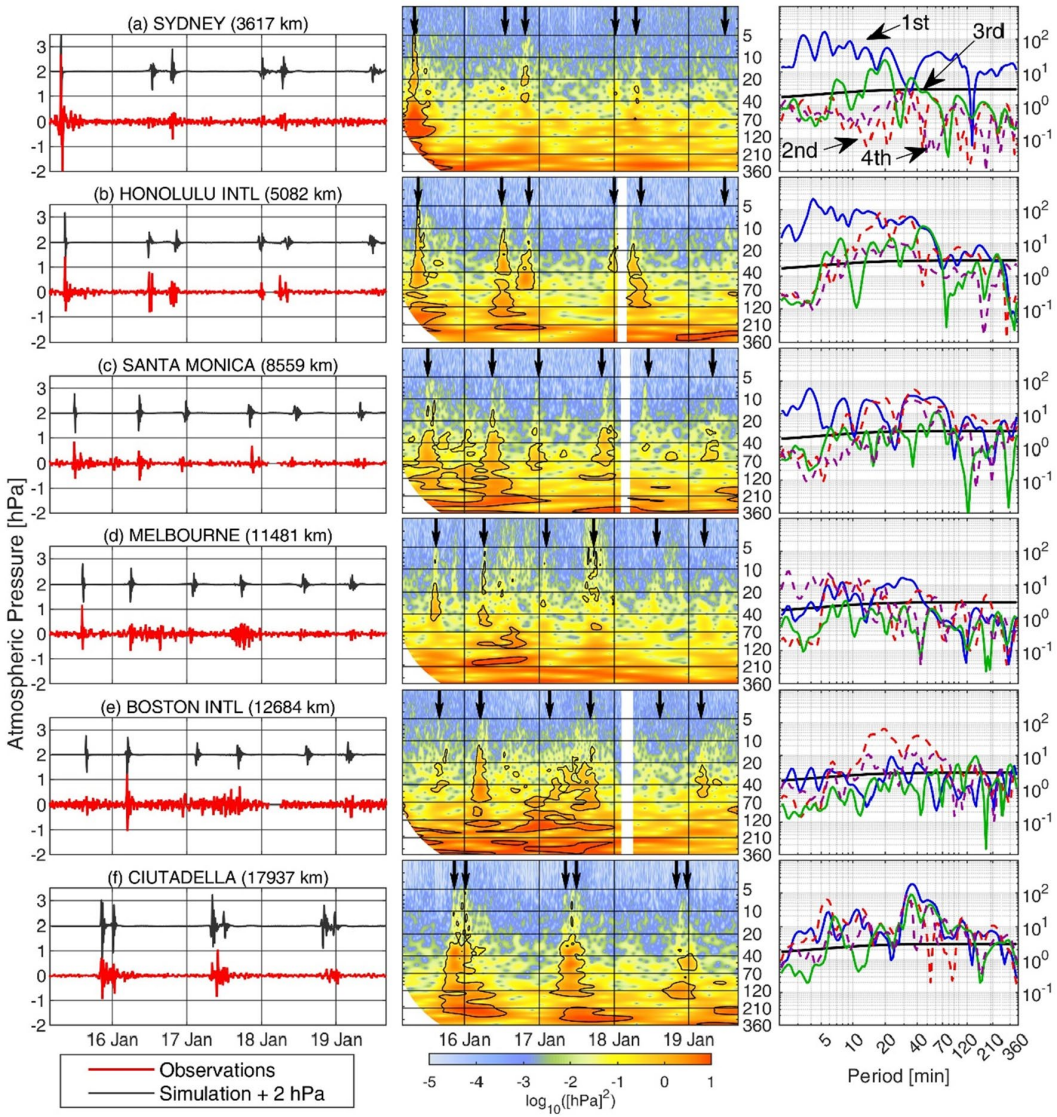
Left panels: high-pass filtered (cut-off period of 2 h) atmospheric pressure time series (in hPa) spanning the period from 0400 UTC 15th January 2022 to 1600 UTC 19 January 2022 (red lines). The location of the series is shown in Fig. 1, and the distance to Tonga is indicated in each plot. The simulated atmospheric pressure anomaly obtained by Ref.^13^ is also included (black lines) shifted 2 hPa for clarity. Central panels: wavelet power spectrum (in log_10_ (hPa^2^)) of the non-filtered atmospheric pressure series at the same locations. The arrows indicate the arrival time of the different wave passages. Right panels: ratio between the energy spectrum computed for each passage (1st in solid blue; 2nd in dashed red; 3rd in solid green; 4th in dashed purple) and the background red-noise spectrum. The energy spectra have been estimated by averaging the WPS over a 1 h period centered at each passage time. The solid black line indicates the 95% significance level estimated as described in Ref.^23^.

Satellite images show that the pressure perturbation was triggered at Tonga archipelago on 15th January 0415 UTC^12^; from subsequent atmospheric pressure observations it was inferred that it travelled to the antipodes, in Algeria, at a speed of around 311 m/s^13^. Figure 2 (left panels) shows some representative atmospheric pressure records (their geographical location is shown in Fig. 1); most of them clearly show six wave passages during the days after the explosion, the odd ones (1st, 3rd and 5th) corresponding to the wave front travelling from Tonga to the stations, and the even ones (2nd, 4th and 6th) corresponding to the wave front arriving from the antipodes. After those, additional passages could have occurred, but their signature is not clear. The arriving times of the waves coincide with those simulated by Ref.^13^ (black arrows and time series in each of the left panels of Fig. 2), which enables the use of that simulation to estimate the arrival times at locations where no atmospheric pressure observations were available.

**Figure 3:**
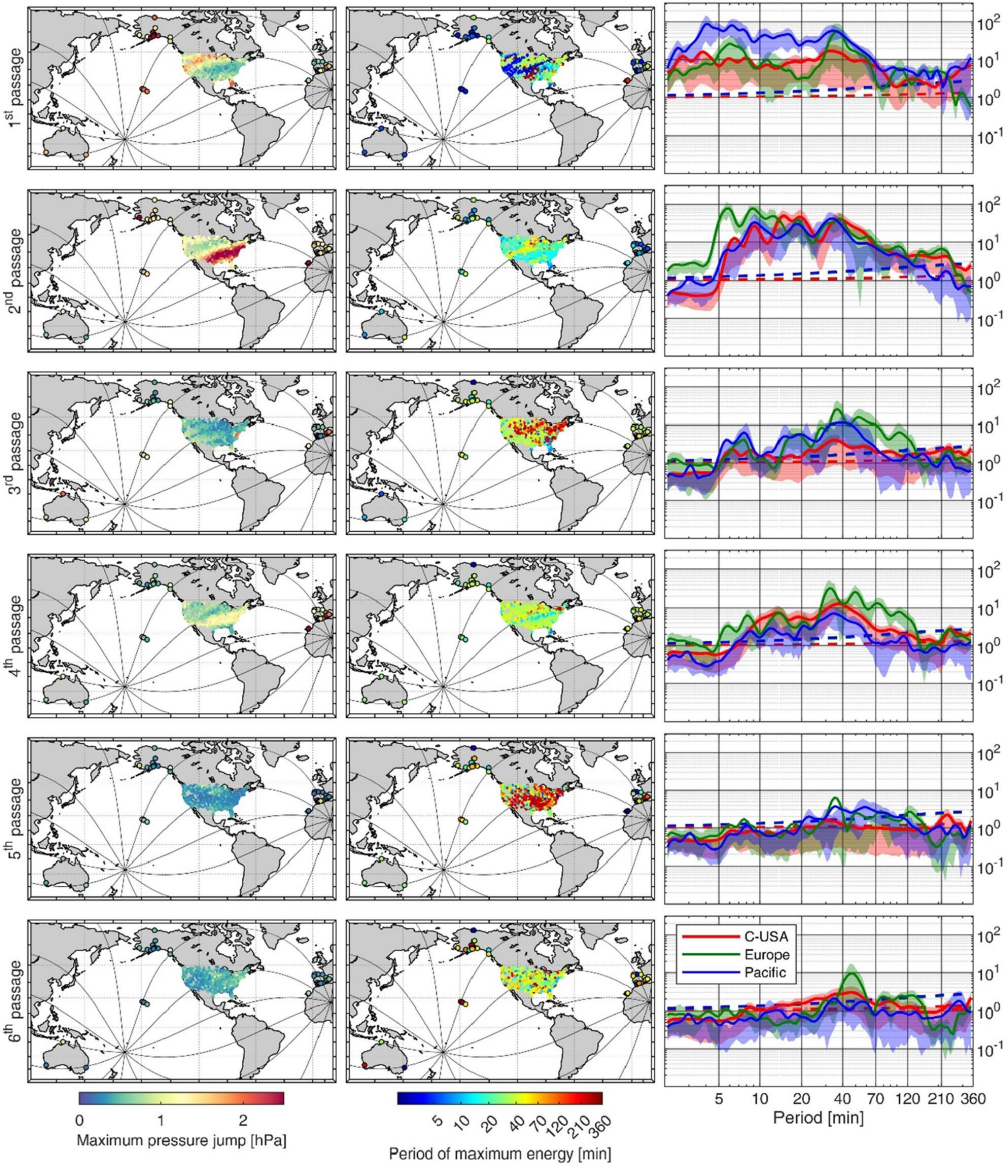
Left panels: maximum pressure jump amplitude (computed as the difference between two consecutive extrema located within a 2 h window centered around the arrival of the atmospheric perturbation) observed at different stations (color dots) and for the different wave passages. The black lines show the trajectory of the wave fronts from Tonga to its antipodes. Central panels: spectral location of the maximum energy increase observed for each station (color dots) and for the different wave passages; it has been determined as the period with the highest ratio between the spectral energy during each passage and the mean (red noise) spectrum of the station. Right panels: regional averages of the ratio between the energy spectra at each wave passage and the background red-noise spectrum computed at each station, as computed for the right panel of Fig. 2. Each panel shows the mean (solid line) and the 75% percentile and the 25% percentile (upper and lower limits of the shadow) for each of the averaged regions (Pacific Ocean, including stations in Australia. Hawaii and Alaska, in blue; continental USA, except Alaska, in red; Europe, in green). The 95% significance level is indicated with dashed lines. The maps have been drawn with MATLAB^33^ using the M_Map^34^ toolbox (https://www.eoas.ubc.ca/~rich/map.html).

The atmospheric wave passages can be identified by large high-frequency oscillations in the pressure time series (Fig. 2, left panels) and by an increase of the energy contents in the high frequency band (below 120 min period) in the Wavelet Power Spectra (WPS, Fig. 2, central panels). The signature of the atmospheric perturbation at different locations shares some similarities, but there are also clear differences in the oscillation amplitudes and in the spectral energy contents among passages and locations. To emphasize this point, Fig. 3 (and SI2) show the maximum pressure jump amplitude (left panels) and the period at which a maximum spectral energy increase is observed (central panels) for each wave passage. The stations represented in the maps of Fig. 3 are located mainly over the continental USA (654 stations located in all States except Alaska), but there are also stations located in the Pacific Ocean (3 stations in Australia, 5 in Hawaii and 12 in Alaska) and in Europe (26 stations).

A plot of special interest to characterize the atmospheric disturbance is the ratio between the spectrum at the time of the wave passages (estimated as a 1-h average of the WPS centered around the passage time) and a background red-noise spectrum (estimated as described in Ref.^23^ and in Methods section). This kind of plot has been produced for each station of Fig. 2 (shown in the right panels of that Figure) and for all the stations of Fig. 3 (in that case the results are presented averaged over the different regions, see the right panels of that Figure).

These Figures show that both the amplitude and the spectral energy distribution are very different for the successive atmospheric wave passage and at different locations. Yet, they also show that all passages caused an increase of the spectral energy content in the high-frequency band (periods < 120 min; see Fig. 3, right panels). It can also be stated that the energy of the atmospheric wave clearly decreased with time, and that the observed heterogeneities between regions and passages are larger than those shown in the idealized numerical simulations available at present (the reader is refereed to Fig. 2 in Ref.^13^, Fig. 3 in Ref.^20^ and Fig. 5 in Ref.^21^).

If we look in more detail to each of the panels of Fig. 3, we observe that during the 1st passage, the energy increase is larger in the Pacific Ocean than in the other regions due to the proximity to the volcano explosion (right panel of 1st row). In Europe and the USA, however, the energy observed during the 2nd passage is higher than during the 1st one, despite the fact that the incoming wave has travelled a larger distance (right panel of 1st and 2nd row). For these two regions, the even passages (2nd, 4th and 6th) systematically present more energy than the odd ones corresponding to the same rotation around the Earth (1st, 3rd and 5th, respectively; Fig. 3 right panels). it is also worth noting that the energy of the highest frequencies (those with periods < 10 min) decreases faster than that for the lower frequencies (Fig. 3, right panels).

Furthermore, the continental USA stations represented in the maps of Fig. 3 (left and central panels) and in Fig. SI2 show a pattern with strong differences between the northwestern and southeastern regions. The pattern is particularly marked for the even passages (which show a higher energy than the odd passages), with the highest energy increase being observed in the southern and eastern USA. No relation has been found between the described pattern of pressure jump amplitudes and the distance traveled by the wave from the source or from the antipodes. No relation has been found either with the topographic barriers crossed by the wave front trajectory.

Finally, it is worth noting that, at locations near the antipodes, the even passages are very close to the odd ones, which results in continuous atmospheric pressure oscillations lasting for more than 3 h (see e.g. Ciutadella, in Fig. 2). This will reflect on the sea level response at these locations, as described in the next section.

### Sea level response to the atmospheric perturbation

**Figure 4:**
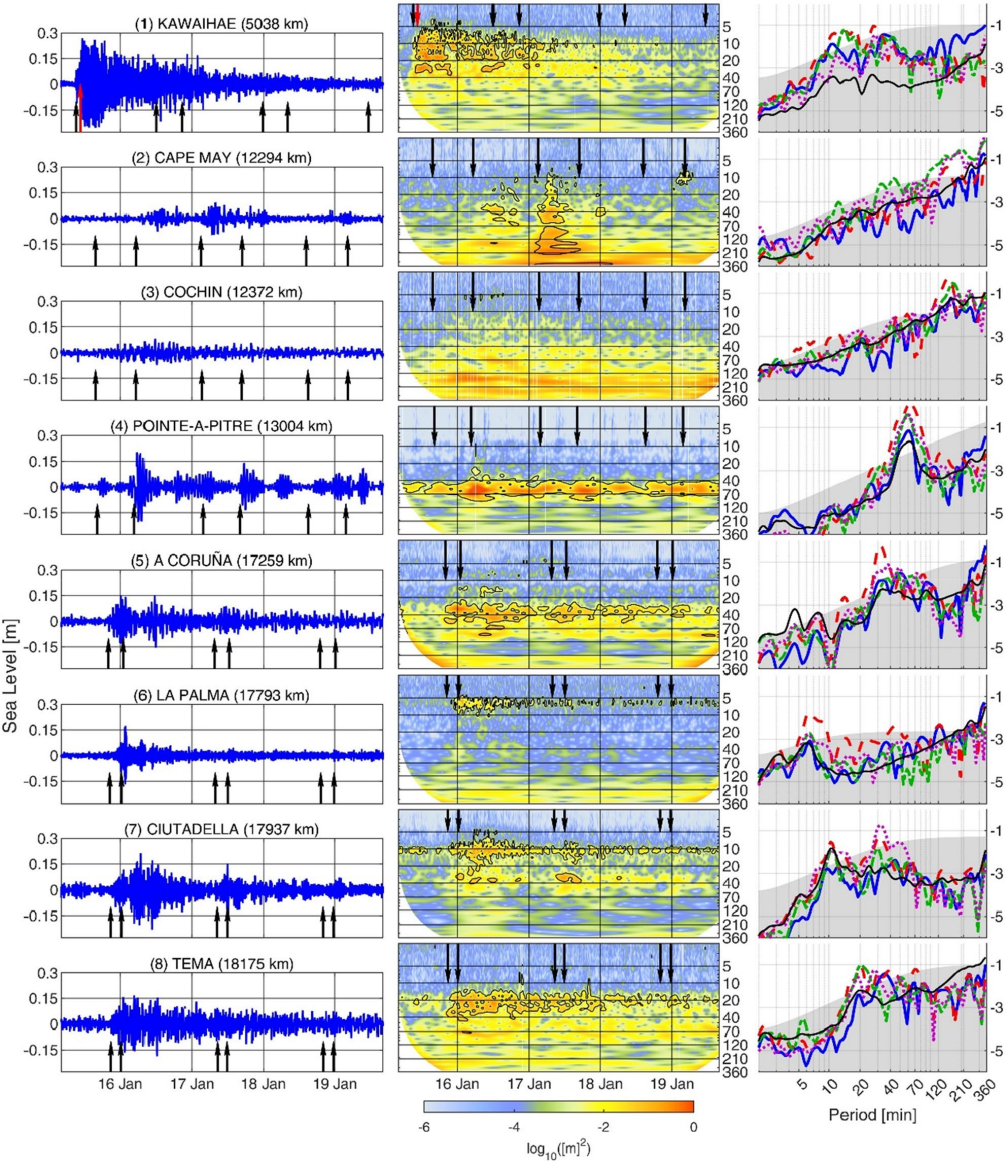
Left panels: high-pass filtered (cut-off period of 2 h) sea level time series (in m) spanning the period from 0400 UTC 15th January 2022 to 1600 UTC 19 January 2022. The location of the stations is shown in Fig. 1, and the distance to Tonga is indicated in each plot. Central panels: wavelet power spectrum (in log_10_ (m^2^)) of the non-filtered sea level series at the same locations. In both, left and central panels, the black arrows indicate the arrival time of the different atmospheric wave front passages, while the red arrow plotted for Kawaihae station, in the Pacific Ocean, shows the arrival time of the tsunami wave. Right panels: spectral energy (in log_10_ (m^2^)) of the SLOs recorded during each passage (1st in solid blue, 2nd in dashed red, 3rd in dashed green and 4th in dashed purple) and the mean spectral energy of the station, computed from a 1-year record (solid black line). The energy spectra have been estimated by averaging the WPS over a 1 h period centered at each passage time. The gray patch indicates the 95% significance level of the colored spectra, which has been estimated as in Ref.^23^ (the significance level is smaller for the background spectra, since it has been estimated by averaging a 1-year time series).

**Figure 5:**
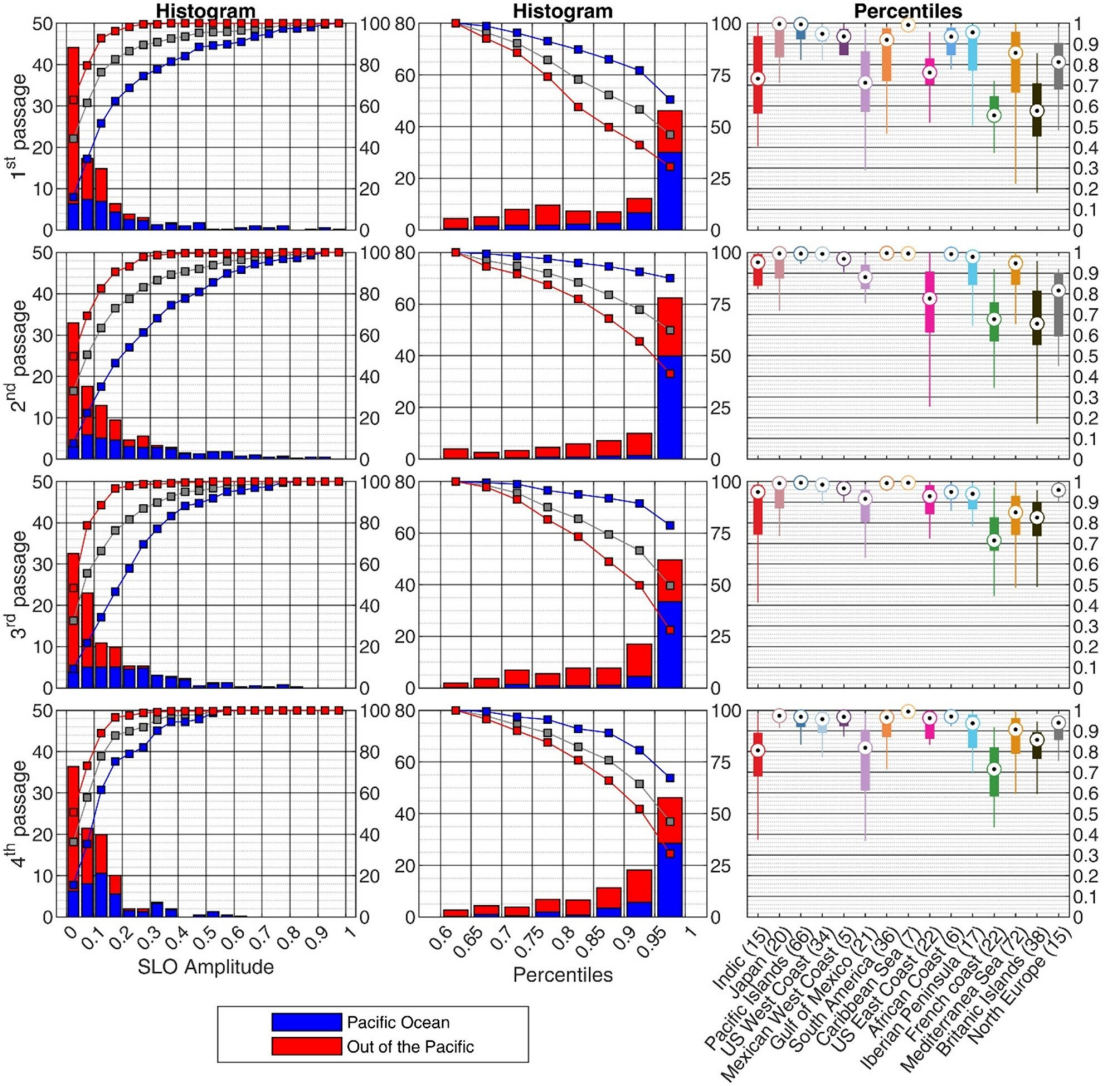
Left panels: probability distribution (in percentage, left scale of the panels) of the maximum SLO amplitudes shown in SI5; the blue fraction of the bars stand for Pacific Ocean tide gauges (with respect to the total number of stations), while the red fraction of the bars stand for tide gauges outside the Pacific (with respect to the total number of stations). The cumulative probability distribution (i.e., the percentage of stations with amplitudes below a given value, right scale of the panels) has been plotted for all tide gauges (in gray), for Pacific Ocean tide gauges (in blue) and for tide gauges outside the Pacific (in red). Central panels: as for the left panels, but for the distribution of the percentiles that the maximum SLO amplitudes observed during the different passages represent in a one-year-long record of SLO amplitudes before the Tonga eruption; in this panel the cumulative probability distribution represents the percentage of stations with amplitudes above a given value, right scale of the panels). Right panels: percentiles averaged within the different world regions defined in Fig. 1 (number of stations in each region is indicated). The boxes represent the Q1–Q3 range of values in each region and the lines represent the range between Q1–1.5(Q3–Q1) and Q3 + 1.5(Q3–Q1), being Q1 and Q3 the 25% and 75% percentiles.

Figure 4 (left panels) shows some representative sea level records (their geographical location is shown in Fig. 1). Tide gauge observations confirmed that the largest SLOs were registered in the Pacific Ocean, where the sea level response to the atmospheric perturbation added to the tsunami wave generated during the volcano explosion. The left column of Fig. 5 shows that in the Pacific Ocean more than 40% of tide gauges measured SLOs larger than 20 cm, and more than 20% surpassed 40 cm during the 2nd passage, which is the one with the largest SLO amplitudes (Fig. 5, left panel of 2nd row). This contrasts with the other basins, where less than 10% of tide gauges measured SLOs larger than 20 cm during that 2nd passage. The percentage is even smaller for the other three passages (Fig. 5 left panels of 1st, 3rd and 4th rows).

As representative of the Pacific Ocean tide gauges, Kawaihae (Hawaii; see the record in Fig. 4) shows the first significant SLOs simultaneously to the arrival of the 1st passage of the atmospheric wave front (denoted with a black arrow), but the maximum amplitudes were observed with the arrival of the tsunami waves generated at Tonga (see the data and methods section for information about the tsunami arrival time estimation; denoted with a red arrow). After that time, SLOs lasted for more than 30 h. This behavior is also observed in most time series of the Pacific Ocean (see Fig. SI3). Disentangling the contributions of the tsunami waves generated by the volcano explosion itself from the meteotsunami waves generated by the atmospheric disturbance is thus difficult, due to the close arrival times of both types of waves to the tide gauges of the Pacific Ocean. For this reason, we will not focus on the Pacific Ocean tide gauges in the study of the global meteotsunami.

Tide gauges outside the Pacific Ocean and therefore far from the area of influence of the tsunami waves also registered SLOs in the tsunami frequency band at times coinciding with the arrivals of the atmospheric wave. This can be observed in the sea level time series of Fig. 4, which show an increase in the amplitude of SLO coinciding with the passages of the atmospheric disturbance (denoted with black arrows). In order to quantify this visual inspection, we computed the ratio between the standard deviation of the filtered time series within two 4-h time windows, one succeeding and one preceding the passages of the atmospheric wave. Thus, a ratio larger than 1.1 would indicate a significant increase in the sea level signal standard deviation of at least the 10% coinciding with the arrival of the atmospheric wave (this threshold is indicated in Fig. SI6 with a dark grey bar). We found that more than 65% of tide gauges showed a ratio greater than 1.1 after the 1st or the 2nd passages; the percentages were much smaller (between 50 and 40%) for the successive passages (see Fig. SI6), indicating a decreasing impact of the atmospheric disturbance. This result, added to the visual inspection of the time series, provides convincing evidence that the atmospheric disturbance generated by the Tonga Volcano explosion generated SLO oscillations in a majority of the tide gauge stations distributed worldwide.

Regarding the magnitude of the generated SLO, during the second passage (the one presenting larger amplitudes worldwide) approximately 30% of the records show amplitudes above 10 cm (Fig. 5, left column of 2nd row), with only a few records exceeding 30 cm, primarily located in the Western Mediterranean, the Caribbean Sea and the African Coast (see Fig. 5 and SI5). For the 1st, 3rd and 4th passages only between 20 and 25% of the tide gauges outside the Pacific Ocean show SLO amplitudes above 10 cm (Fig. 5, left column).

**Figure 6:**
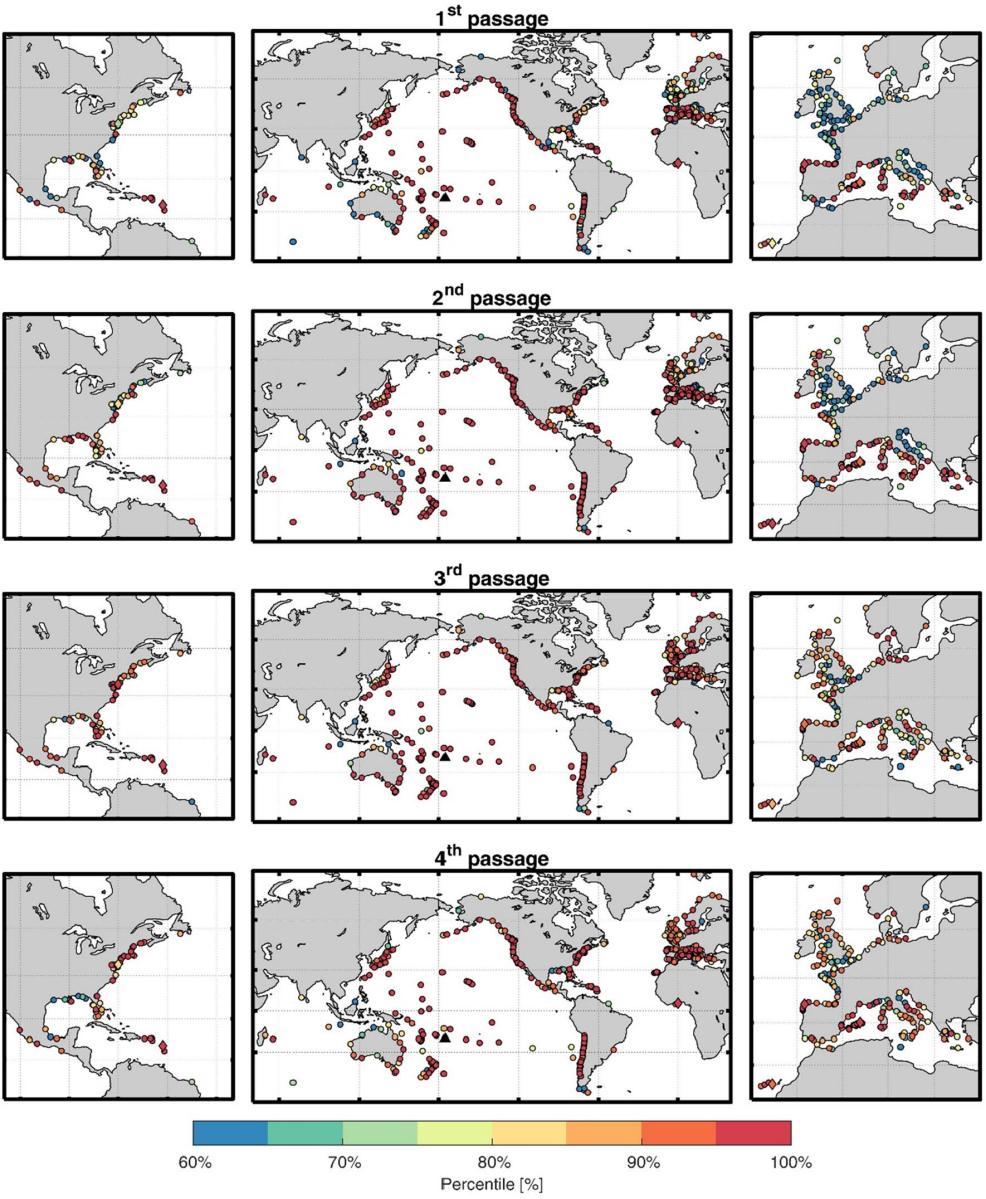
Map of the percentiles that the maximum SLO amplitudes observed during the different passages (shown in SI5) represent in a year-long record of SLO amplitudes before the Tonga eruption. The central panels show the whole globe, while the left and right panels are zooms on the East Coast of USA and Europe, respectively, where the density of observations is higher. The stations with less than one year of data (and therefore preventing the computation of percentiles) are not shown. The location of the time series plotted in Fig. 4 are marked with a diamond. The maps have been drawn with MATLAB^33^ using the M_Map^34^ toolbox (https://www.eoas.ubc.ca/~rich/map.html).

The value of the maximum SLO observed at every station is not necessarily a good indicator of the impact of the Tonga atmospheric disturbance, as there can be stations routinely submitted to different forcings and therefore having a different variability background. For the sake of comparison with the background variability, we computed for each tide gauge the percentile that the maximum SLO amplitude observed during the event represents within a year of measurements obtained before the Tonga eruption (see Fig. 6 and the central panels of Fig. 5). Even though the patterns shown in Fig. 6 are very heterogeneous, even for neighboring stations, the SLO amplitudes associated with the 2nd passage of the atmospheric perturbation (the one generating the largest SLOs) correspond to the upper 80-percentile for a 75% of tide gauges located outside the Pacific Ocean (Fig. 5, central panel of 2nd row). For more specific regions (those defined in Fig. 1), the percentile analysis shows that in the Pacific Ocean regions (Japan, Pacific Islands, USA West Coast, Mexican West Coast and South America) the SLOs recorded during the passage of the Tonga perturbation are in the upper 90-percentiles for most tide gauges (see Fig. 5, right panels). Outside the Pacific Ocean the response is more heterogeneous (see the dispersion bars for each region): although the mean values of all regions keep above the 70-percentile, smaller percentiles (below 50%) are obtained at some stations of the Gulf of Mexico, Indic Ocean and the French Coast. Summarizing, the percentile analysis shows that in most tide gauge stations the SLO amplitudes resulting from the Tonga eruption were larger than the background variability observed at the station (i.e., the observed amplitudes fall within the upper percentiles), although they did not represent historical maxima anywhere.

It is worth noting that some of the SLO values used in the percentile analysis might not be forced by the atmospheric disturbance generated by the Tonga Volcano eruption. The simultaneity between the onset of the SLOs and the arrival of the atmospheric wave at every station (illustrated in Fig. 4 for selected stations and in SI6) surely corresponds to a cause-effect in most cases, but not necessarily in all them. In the East Coast of the USA, for instance (represented by Cape May station in Fig. 4), no significant response was detected during the 1st passage (when the perturbation arrived from inland and was very weak), while during the even passages (when the perturbation arrived from the ocean) SLOs appeared a few hours after the passages. For the 3rd passage, which could be expected to be similar to the 1st one, Cape May and other stations located in the same region show the largest impact, compared with the other passages (Fig. 6 and SI5, left panels). However, the USA National Weather Service reported the occurrence of a strong storm along the USA East Coast at the time of the 3rd passage^24^. This weather system left its signature at the atmospheric pressure time series of nearby stations (Melbourne and Boston, Fig. 2). Therefore, the SLOs observed in the region during that passage can be attributed to a meteorological phenomenon not related to the Tonga eruption. A similar feature seems to happen also during the 3rd passage of the atmospheric wave over northern Europe. In spite of these exceptions, the evidences that the passage of the atmospheric disturbance generated a global meteotsunami can be considered unequivocal.

Other tide gauges also show differences between odd and even passages, but without any delay between the arrival of the atmospheric wave and the SLOs (see e.g. Pointe-à-Pitre, Guadalupe, in Fig. 4, chosen as representative of the Eastern Caribbean Islands). At stations closer to the antipodes, SLOs triggered during the odd passages are observed to last until the arrival of the even passages, a few hours later, which added energy to the remaining oscillations and resulted in larger amplitudes. In fact, at tide gauges such as A Coruña (in the Atlantic coast of the Iberian Peninsula) or Ciutadella (in the Balearic Islands, Western Mediterranean Sea), the largest SLOs were registered a few hours after the two consecutive wave front passages.

The differences in the oceanic response to the atmospheric perturbation are more marked when looking at the spectral energy observed in different tide gauge records. The spectral analysis carried out for sea level time series is similar to that of atmospheric pressure time series shown in Fig. 2: the time-dependent spectral energy distribution (the WPS) of the selected tide gauges is shown in the central panels of Fig. 4. The right panels of Fig. 4 show the spectral energy of the SLOs recorded during each passage and the mean spectral energy of the station (computed from a 1-year record) themselves, not their ratio as in Fig. 2. These plots reveal a key feature: the maxima of the sea level spectral energy observed during the atmospheric wave passage are collocated with the eigenperiods identified in the mean spectrum (black lines in the right panels of Fig. 4) and are therefore particular of each station.

Two examples are paradigmatic; on one hand, Pointe-à-Pitre (Eastern Caribbean Islands) has an eigenperiod at about 50 min (Fig. 4, right panel of 4th row), which falls within the frequency band at which the atmospheric disturbance showed a maximum energy increase over the USA (Fig. 3, right panels). As expected, the spectral energy of the SLOs observed at that station has a marked maximum around this period (Fig. 4, right panel of 4th row). On the other hand, Ciutadella (Western Mediterranean), one of the stations outside the Pacific Ocean that registered the largest amplitudes, shows 3 eigenperiods: a main one at 10 min and two secondary ones at about 24 and 33 min (Fig. 4, right panel of 7th row). Since 10 min is somehow at the edge of the frequency band where the atmospheric disturbance shows a maximum energy increase (particularly after the 2nd passage, due to the faster dumping of the highest frequencies), the spectra measured during the atmospheric wave passages do not show a marked increase at 10 min either, when compared with the mean spectrum. Even so, the 10 min SLOs observed at Ciutadella are relevant, because so it is the energy content at this period. Conversely, the other two eigenperiods of Ciutadella show a larger energy increase when compared with the mean spectrum, but do not always result in energies larger than those at 10 min, because in absolute terms these modes not always have energy contents larger than those of the main mode (Fig. 4, right panel of 7th row).

## Discussion

The aim of this section is to infer the main physical processes underlying the observed characteristics of the global meteotsunami generated by the Tonga volcano eruption. In particular, we intend to link the major features of the atmospheric pressure and sea level records commented in the previous sections, and to explain the large heterogeneity observed in the sea level response.

The analysis of globally distributed atmospheric pressure records has evidenced that the energy of the atmospheric waves generated by the explosion of the Tonga volcano was concentrated in the tsunami frequency band (from 2 to 120 min). The atmospheric perturbation circled the Earth at least three times, triggering an oceanic response that was recorded at many locations around the world, in what can be called a global meteotsunami. The magnitude of the ocean response was only hazardous in the Pacific Ocean, where SLOs were produced by different sources and not only by the atmospheric disturbance, as explored in other studies^15–18,20,21^. Therefore, the singularity of this event does not rely on the large amplitude of the observed SLOs, but on the fact that a distant volcano explosion generated a noticeable sea level response worldwide.

As stated repeatedly along this work, a key feature of the global meteotsunami was its spatial heterogeneity. A first source of differences can be the characteristics of the atmospheric disturbance as observed at different locations. As commented above, the geographical distribution of the maximum atmospheric pressure jump detected by the barographs shows well defined patterns even at regional scale (e.g. over North America, see the left panels of Fig. 3). No general explanation has been found so far for the observed patterns. Other differences have an easier explanation: near the antipodes, the even passages of the atmospheric wave are very close to the odd ones, which results in continuous atmospheric pressure oscillations lasting for several hours over the Western Mediterranean, for instance (see e.g. the Ciutadella record in Fig. 2).

Regarding the characteristics of the oceanic response, a first consideration to be made is that although the atmospheric pressure oscillations fluctuated between 1 and 3 hPa, depending on the observation site, the SLO amplitudes were of a few tens of cm at many locations. Thus, some amplification mechanism is required^1^, since the direct static response to the atmospheric pressure variations only accounts for one cm per hPa (the so-called inverted barometer response). There are several physical mechanisms that can amplify the sea level response of an atmospheric disturbance. The Proudman resonance, i.e. the coupling between the atmospheric disturbance and the associated free oceanic wave^25^, is usually crucial in meteotsunamis^1^. It requires the propagation speed of the atmospheric perturbation to be close to the speed of the oceanic free wave, which depends on the ocean bottom depth ($c=\sqrt{gh}$, where *g* is the gravity acceleration and *h* is the ocean depth). The novelty of the case studied here with respect to meteotsunamis generated by weather systems is that the atmospheric waves propagated at a speed of ~ 311 m/s; therefore, Proudman resonance would only be relevant at locations with ocean depths between 8200 and 12,000 m^26^. According to the simulations of Ref. 15, 16 and 22, the maximum amplification produced by this mechanism would have been of ~ 1.5 in the Pacific Ocean, where the mean depth is ~ 6000 m. In other, shallower oceans, the amplification would have been even smaller, since the propagation speed of the atmospheric perturbation is much larger than the velocity of ocean free waves.

The numerical simulations carried out by Refs.^21,22^ show that waves of a few cm were generated in the open ocean during the passage of the atmospheric disturbance. The simulations also show that these waves were organized in several wave fronts, since when the atmospheric wave overtakes the ocean waves the latter are decoupled and travel at a lower speed as tsunami waves^16,21^. The generation of different wave fronts explains the long duration of the SLOs measured by many tide gauges (Fig. 4, left panels), since they result in a continuous arrival of waves from the open ocean to the coast. As the waves arrive at the coast, they could be amplified by a factor of 2–4 due to shoaling effects, according to Green’s Law^27^.

Shelf waves can also be triggered by the atmospheric perturbations during its pass over the continental shelves^28,29^. Reference^9^ analyzed the SLOs produced after the Krakatoa explosion and suggested that an amplified ocean free wave is generated when a supercritical atmospheric disturbance (i.e., a perturbation moving faster than the oceanic free waves) crosses a continental shelf step on its way towards the coast. Namely, the oceanic wave is released at the shelf edge and travels slower than the atmospheric perturbation, causing a time lag between the recording of the atmospheric perturbation and the recording of SLOs at the coast. The amplification factor depends on both the ocean and shelf depths. According to Ref.^9^, in the case of the USA eastern continental shelf the amplification factor would be around 2. Moreover, the shelf of the East coast is wide enough (100–400 km) as to observe a delay between the passage of the atmospheric perturbation and the SLOs of a few hours, which is consistent with the SLOs observed at Cape May during the 2nd atmospheric wave passage (Fig. 4, left panel of 2nd row). Shelf edges also produce a wave reflection that confines the energy near the coast, preventing the leakage of the shelf waves energy towards the open ocean. This would explain the long duration of SLOs observed at locations such as A Coruña or Tema, in the Atlantic Ocean (see Fig. 4, left panels) or in the Balearic Island, in the Western Mediterranean (see Fig. SI4).

Nevertheless, the differences observed between stations very close to each other must be attributed to other, local processes, rather than to regional processes. Namely, harbour or bay resonance, which occur when the frequencies of the impinging waves are close to the eigenmodes of the harbour or bay^30^, must play an important role. In fact, the spectral analysis of tide gauge records shown in Fig. 4 (right panels) revealed that the energy is concentrated around the eigenperiods of each station. The cases of Pointe-à-Pietre and Ciutadella, commented in the previous section, would support this hypothesis.

Also continental shelves have oscillation eigenmodes that can amplify some frequencies and cancel out others. Outside Ciutadella, for instance, Ref.^31^ measured energetic SLOs excited by an atmospheric pressure anomaly with peaks around 30 min. This is in agreement with the two secondary eigenperiods appearing in the mean spectra of that station, shown in the right panel of the 7th row of Fig. 4 (the main eigenperiod at 10 min corresponds to the fundamental mode of the inlet). Finally, it is worth mentioning that both harbour and shelf resonance result in a maximum amplification when a continuous pumping of energy exists^30^. The long-lasting atmospheric oscillations observed at stations near the antipodes could thus explain why some of the largest SLOs outside the Pacific Ocean are observed by tide gauges close to the antipodes.

Summarizing, the meteotsunami generated by the Tonga eruption is unique for two major reasons: for its global dimension and because the triggering atmospheric disturbance traveled at a speed ten times larger than the atmospheric gravity waves or the convective systems that trigger more common meteotsunamis. The spatial heterogeneity of the sea level response observed worldwide would be due to several causes. First, to differences in the forcing atmospheric wave, as observed at different locations, which in turn would be due to the station location and to other, no resolved features. Most importantly, the sea level response would be modulated by the dimensions and bathymetry of the continental shelves and of the harbours where tide gauges are located. The eigenmodes of both, shelves and harbours would act as tuners for the surface waves generated by the atmospheric disturbance at open sea, amplifying the signal mainly at their eigenperiods and cancelling out other frequencies.

In order to explain the sea level response observed at each location, a deeper analysis of each ocean basin, including shelf and harbour geometries, would be required. The proper way to do it would be using regional high-resolution numerical simulations with very high-resolution bathymetries near the coast. This is still a pending work, since the global simulations published so far are very useful to understand the generation of the open sea waves, but do not have the required resolution to resolve all the described regional and local features.

## Methods

### Atmospheric pressure data

The atmospheric pressure data used in this work come from the NOAA Automated Surface/Weather Observing System (ASOS/AWOS, 889 stations in the United States), the Spanish Meteorological Agency (AEMET, 230 stations in Spain), the Spanish Holding of Harbours (Puertos del Estado, 19 stations in Spain), the Balearic Coastal Observing and Forecasting System (SOCIB, 6 additional stations in the Balearic Islands, Western Mediterranean), PortsIB (1 station at Ciutadella, also in the Balearic Islands), and the Australian Bureau of Meteorology (3 stations). All time series cover the period 15–21 January 2022 and have a sampling interval of 1 min except AEMET stations, which have a sampling interval of 10 min. Only stations with less than 10% of missing values were used (894 stations out of 1129).

### Sea level data

Sea level data were obtained from the Intergovernmental Oceanographic Commission (IOC, 732 tide gauges distributed worldwide), the NOAA Center for Operational Oceanographic Products and Services (CO-OPS, 19 tide gauges in the USA), the Service Hydrographique et Océanographique de la Marine (SHOM, 110 tide gauges in France and old overseas French territories), Puertos del Estado (30 tide gauges in Spain), SOCIB (6 tide gauges in the Balearic Islands) and PortsIB (1 tide gauge in Ciutadella, Balearic Islands). The length of the retrieved time series is of at least one year and all series cover the period 1–31 January 2022. The time resolution of the observations is 1 min for most of the stations and less than 15 min for all of them. After discarding tide gauges repeated in different datasets, only stations with less than 10% of missing values between 15th and 21st of January 2022 were used. Finally, after visual inspection, some tide gauges showing jumps or inconsistent records were also eliminated, leaving a total of 472 out of 898 sea level records.

The sea level records shown in Fig. 4 were considered to be representative of a world region, but it must be kept in mind that a key feature of the meteotsunami was its high spatial heterogeneity, and therefore significant differences can exist between nearby tide gauges. The selection was performed after the inspection of the vast amount of available observations and intended to illustrate the different casuistic underlying the sea level response observed worldwide.

### Data analysis methods

The Wavelet Power Spectrum (WPS) was used to study the spectral energy distribution of atmospheric pressure and sea level time series. The spectra and their 95% confidence levels were computed from the original time series using the Ref.^23^ codes, using zero padding, a Morlet wavelet, $\updelta j=1/16$ and ${\upomega }_{0}=12$. The time series appearing in the left panels of Figs. 2 and 4 result from a high-pass filtering of the original time series performed with a second-order Butterworth filter with a cut-off period of 2 hours^32^, in order to highlight the tsunami frequency band.

The timing of each atmospheric wave front passage was computed from the numerical simulation described in Ref.^13^, determining the peaks in the function resulting from averaging the absolute value of the simulation outcome within a 1-h moving window. The arrival time of the tsunami wave to the Pacific Ocean tide gauges was estimated assuming a wave speed $c=\sqrt{gH}$, being $H$ the mean ocean depth along the path from Tonga to the tide gauge. For the right panels of Figs. 2 and 3, we calculated the ratio between the spectral energy during each passage (estimated as a 1-hout time average of the WPS centered at each passage time) and the background red-noise spectrum of each station. The background red-noise spectrum was estimated using Eq. 16 in Ref.^23^ and with the variance and the lag-1 autocorrelation lag computed from the atmospheric pressure time series measured during January 2022. For the right panels of Fig. 4, the spectral energy during each passage was estimated as for Figs. 2 and 3, while the mean spectrum was obtained from a 1-year record.

The amplitude of the oscillations was computed as the difference between two consecutive high/low local extrema in the filtered time series. A time series of SLO amplitudes with a resolution of 1 h was built up for each station, selecting the maximum amplitude within a 1-h moving window for the whole period of available observations. From that SLO amplitude time series we selected the maximum value corresponding to each atmospheric perturbation passage within a four hours window starting one hour before the passage and ending three hours after the passage (the timing of the passage is obtained from the simulated atmospheric signal as described in the previous paragraph). The asymmetry of the window with respect to the passage time intends to contemplate the possibility of some delay between the passage of the atmospheric disturbance and the arrival of free oceanic waves generated upstream. This maximum SLO amplitude is taken as representative of each passage in every tide gauge (see SI5). Otherwise, the amplitudes of the atmospheric disturbances plotted in the left panels of Fig. 3 were obtained in a 2-h window centered at each passage. Finally, for the maximum SLO amplitude observed during each of the passages we found the equivalent percentile in the SLO amplitudes distribution of each tide gauge (Fig. 5, central and right panels, and Fig. 6). This distribution was obtained from a one-year-long SLO amplitude time series recorded before the Tonga eruption. In this way the percentile analysis compares the SLO observed during the Tonga event with an estimate of the background variability of each station.

## Supplementary Information

Supplementary Information.

## Data Availability

The data used in this work are freely available in the following sites: NOAA 6 min Sea Level: https://tidesandcurrents.noaa.gov/stations.html?type=Water+Levels; NOAA CO-OPS Sea Level: https://www.ngdc.noaa.gov/hazard/tide/; NOAA Atmospheric Pressure: https://mesonet.agron.iastate.edu/request/asos/1min.phtml#; SOCIB: https://www.socib.es/?seccion=observingFacilities&facility=mooring; Puertos del Estado: https://www.puertos.es/es-es/oceanografia/Paginas/portus.aspx; SHOM: https://data.shom.fr/donnees/refmar/126/download; IOC: http://www.ioc-sealevelmonitoring.org/list.php; DART:https://www.ngdc.noaa.gov/hazel/view/hazards/tsunami/related-runups/5824.

## Code availability

The Matlab^33^ scripts used to analyze the data and elaborate the figures are available from the authors. We use the Matlab Toolbox created by Ref.^23^ to compute the WPSs (https://atoc.colorado.edu/research/wavelets/). The maps were plotted with the M_Map toolbox^34^ (https://www.eoas.ubc.ca/~rich/map.html).
