# Supplementary material for: Observational study of the heterogeneous global meteotsunami generated after the Hunga Tonga–Hunga Ha’apai Volcano eruption
Source: Sci Rep. 2023 May 27;13:8649. doi: 10.1038/s41598-023-35800-6 (PMC10224910; doi:10.1038/s41598-023-35800-6)
Supplement: Supplementary file 1 — Supplementary Information. [file 41598_2023_35800_MOESM1_ESM.pdf]

Observational study of the heterogeneous global meteotsunami generated after the Hunga Tonga – Hunga Ha'apai Volcano eruption

Joan Villalonga*[1,2,3], Àngel Amores[2], Sebastià Monserrat[2], Marta Marcos[2,3], Damià Gomis[2,3], Gabriel Jordà*[1]

[1] Centre Oceanogràfic de Balears, CN-Instituto Español de Oceanografía (IEO-CSIC), Palma, Spain
[2] Departament de Física, Universitat de les Illes Balears (UIB), Palma, Spain.
[3] Institut Mediterrani d'Estudis Avançats (UIB-CSIC), Esporles, Spain.

Email: joan.villalonga@ieo.csic.es, gabriel.jorda@ieo.csic.es

Supplementary information

SI1: Non exhaustive compilation of news about the Tonga volcano eruption

Social networks:

- https://twitter.com/JoanVLlauger/status/1486704758870134784
- https://twitter.com/imthursty/status/1482816524490579971
- https://twitter.com/MathewABarlow/status/1482528518147825665
- https://twitter.com/an_amores/status/1484516695087759363
- https://twitter.com/JDiazCustatussi//1484215002399707138
- https://twitter.com/diegoaliaga2/status/1486942045524705281
- https://twitter.com/metoffice/status/1482605906659622914?t=yd08VZuIczkCn7Jkc5hzhQ&s=08
- https://twitter.com/AGU_Eos/status/1484654467253428227
- https://twitter.com/TransTerraScape/status/1482704383532224516
- https://twitter.com/akrherz/status/1482436390105272320?s=20
- https://twitter.com/ChurchillWx/status/1482262764924014592
- https://twitter.com/BastiBrings/status/1482806925985206275
- https://twitter.com/weather_models/status/1483006436750028801

Youtube:

- https://www.youtube.com/watch?v=GNmsV4nJoI0
- https://www.youtube.com/watch?v=KsvlrfKevZg
- https://www.youtube.com/watch?v=9LzMaPzYZSM
- https://www.youtube.com/watch?v=7W3rwuTdi7Y

Others:

- https://www.eumetsat.int/hunga-tonga-hunga-haapai_2022
- https://mainichi.jp/english/articles/20220117/p2a/00m/0sc/021000c

- https://earthobservatory.nasa.gov/images/149474/tonga-volcano-plume-reached-the-mesosphere
- https://thedocs.worldbank.org/en/doc/b69af83e486aa652d4232276ad698c7b-0070062022/original/GRADE-Report-Tonga-Volcanic-Eruption.pdf
- https://volcano.si.edu/volcano.cfm?vn=243040
- https://volcano.si.edu/showreport.cfm?doi=GVP.WVAR20220112-243040
- https://matangitonga.to/2022/01/15/tongan-geologists-stunning-Jan14eruptions
- http://global.weathernews.com/news/16551/
- https://www.facebook.com/tongageologicalservice

SI2: Zoom of the pressure anomaly maps

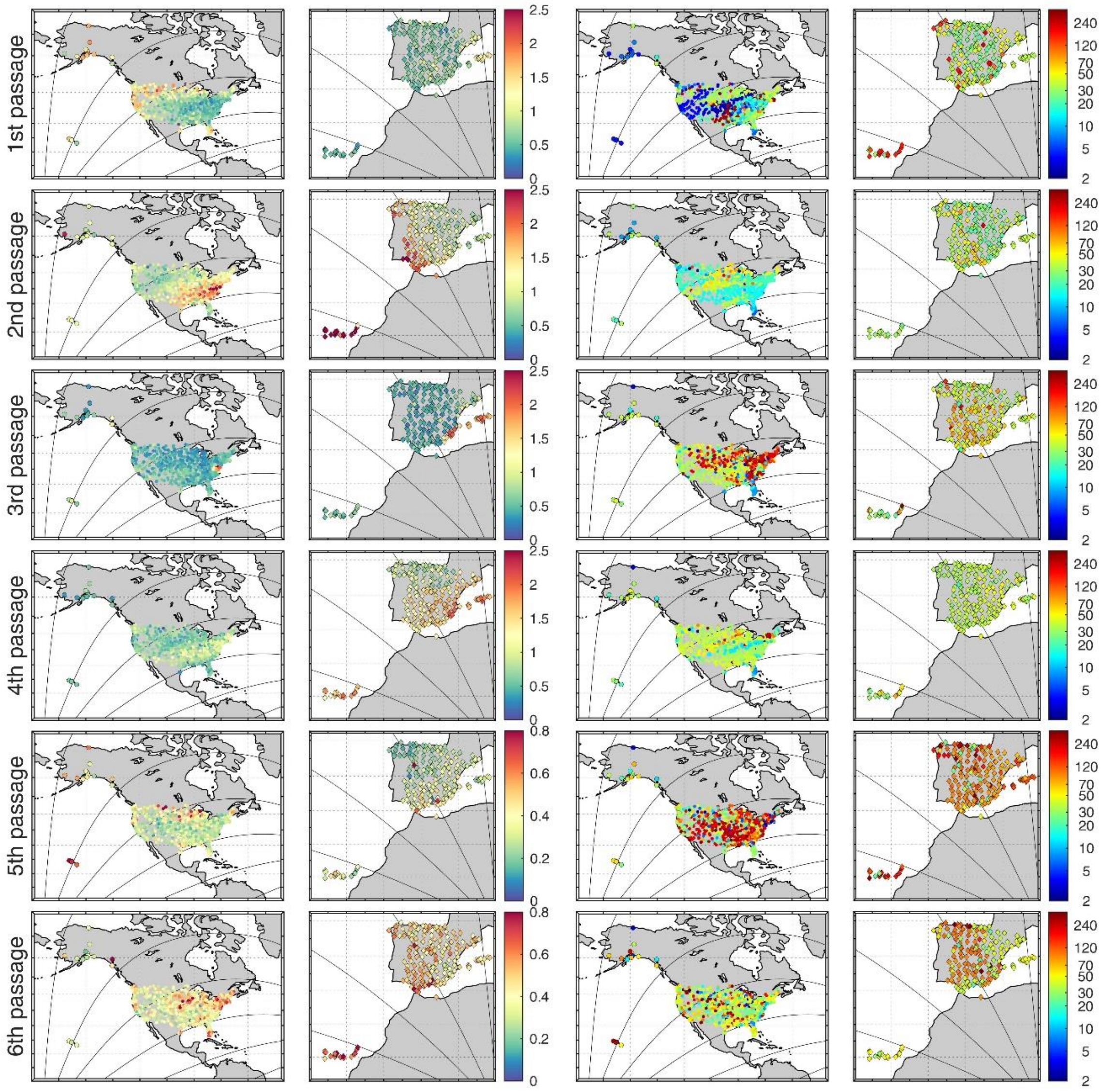

SI.2: Zoom over USA and Spain of Fig. 3. Maximum pressure jump amplitude computed as the difference between consecutive extrema in a 2 hours window centered around the arrival of the atmospheric perturbation (left panels) and period of maximum spectral energy increase, selected as the period with maximum in the spectral ratio between the spectral energy at each passage and the red noise spectrum of the stationat each barograph station for each wave pass , (right panel). Most of the stations (circles) have 1 minute of sampling period but the AEMET ones (diamonds) have 10 minutes of sampling period. In the left panel all time series have been homogenized to a 10-min temporal resolution to get comparable pressure jumps amplitude. This have not been done in the right panels, where it should be taken into account that the maximum in energy in the AEMET stations can not be detected under 10 min period due to the lack of temporal resolution of this stations. The black lines show the trajectory of the wave fronts from Tonga to its antipodes. The maps have been drawn with MATLAB[31] using the M_Map[32] toolbox (https://www.eoas.ubc.ca/~rich/map.html).

SI3: Effects of the vulcano induced tsunami in the Pacific Ocean.

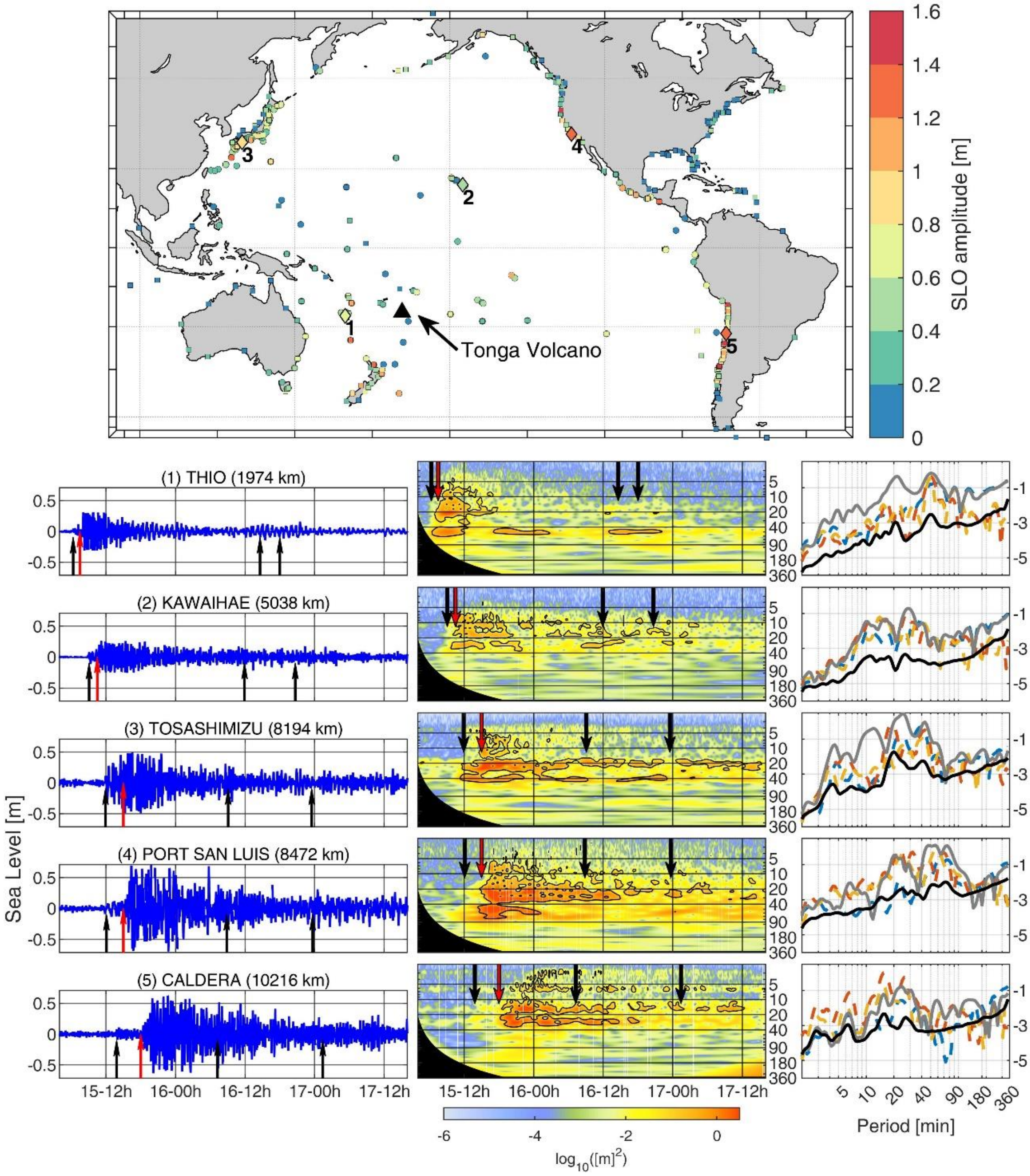

SI. 3: (Top) Maximum tsunami SLOA map from DART reports (circles) and from NOAA, SHOM and IOC time series (squares). The maximum SLO amplitude have been computed as the difference between consecutive extrema in the sea level time series between 0400 UTC 15th January and 1600 UTC 19th January. (Bottom) Time series (right) and corresponding WPS (center) for several stations in the Pacific Ocean between 0400 UTC 15th January and 1600 UTC 17th January. The black arrows indicate the arrival time of the different atmospheric wave passages while the red arrows indicate the arrival of the tsunami wave. The left panel shows spectral energy (in $\log_{10}$ (m^2)) of each passage (1st in blue, 2nd in red and 3rd in yellow) and during the tsunami wave arrival (gray solid line) and the mean spectral energy over a 1-year (solid block line). The spectral energy has been computed by time averaging the WPS with a 1-hour window centered in the atmospheric wave passages or in the time of arrival of the tsunami wave and for 1 year for the mean spectrum. The map has been drawn with MATLAB[33] using the M_Map[34] toolbox (https://www.eoas.ubc.ca/~rich/map.html)

SI4: Sea level and atmospheric pressure records in the Balearic Islands (Western Mediterranean)

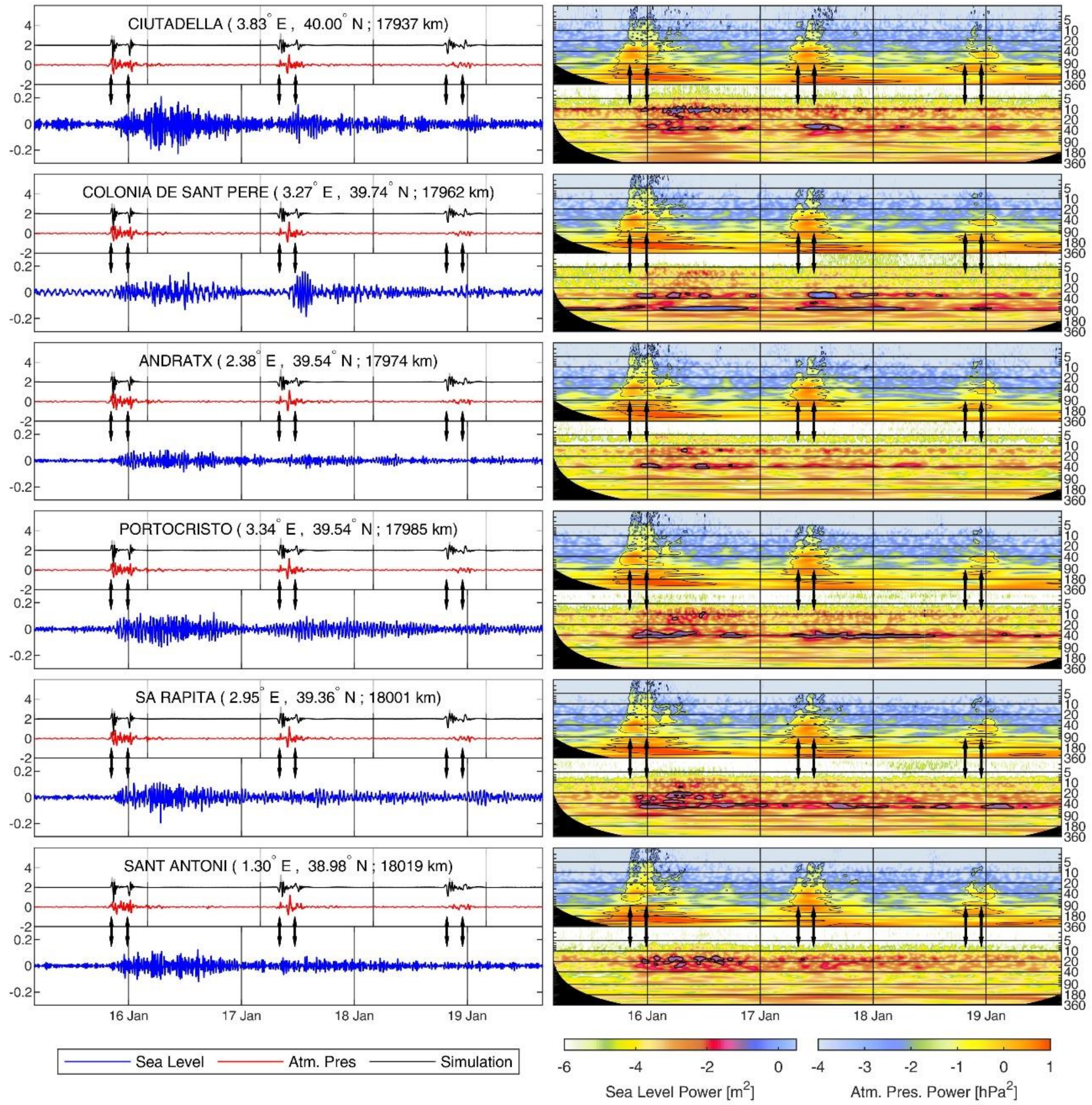

SI. 4: (Left) Filtered time series of the sea level (in m, blue) and atmospheric pressure (in hPa, red) and (right) their corresponding WPS at different locations in the Balearic Islands (western Mediterranean) between 0400 UTC 15th January and 1600 UTC 19th January 2022. The black arrows indicate the arrival time of the different wave front passes.

SI5: Map of maximum SLO amplitudes observed during the different passages

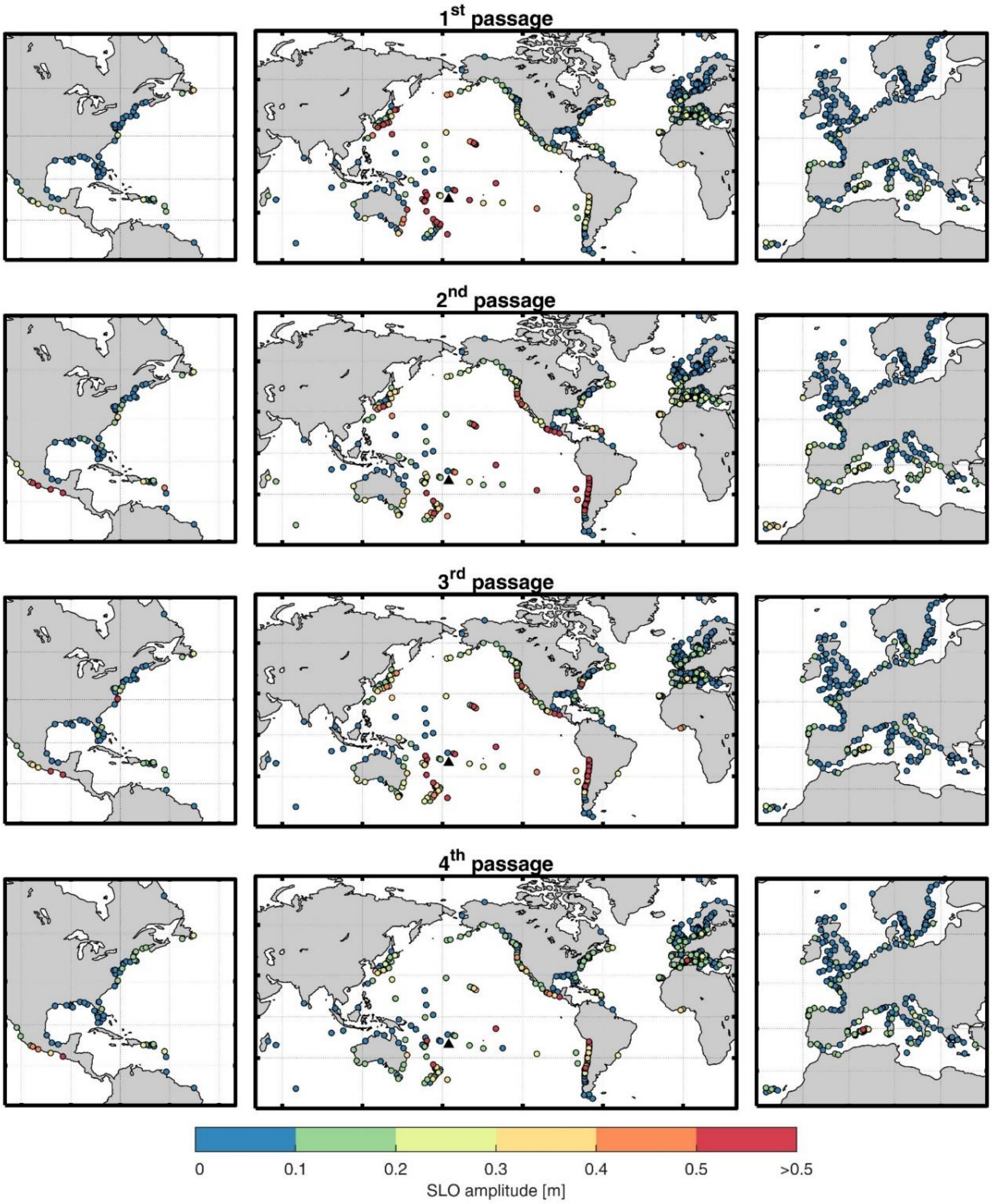

SI.5: Map of maximum SLO amplitudes, computed as the difference between two consecutive extrema located within a 2-hour window spanning from 30 min before the arrival of the atmospheric perturbation to 90 min after. The central panels shows the whole globe, while the left and right panels are zooms on the East Coast of the USA and Europe, respectively, where the density of observations is higher. The maps have been drawn with MATLAB33 using the M_Map toolbox (**https://www.eoas.ubc.ca/~rich/map.html**).

SI6: Analysis of the increase of SLO amplitudes during the atmospheric wave passages.

In order to have a quantitative measure of the simultaneity between the passage of the atmospheric disturbance and the triggering of SLO at tide gauge stations, we performed the following analysis. For each station we computed the standard deviation of the filtered sea level time series within two windows, the first one spanning from T0-5h to T0-1h (where T0 is the arrival time of the atmospheric disturbance at each station) and the second one spanning from T0-1h to T0+3h. The ratio between the standard deviation just after and just before the atmospheric wave passage can be considered a measure of the amplification of the amplitude of SLO caused by the atmospheric disturbance. This "amplification ratio" was obtained for each atmospheric wave passage and for every tide gauge station.

Considering that some tide gauges are mostly (or only) triggered by the even passages and others are triggered by odd passages (see Figs. 4, 5 and 6), we selected the maximum amplification ratio between every couple of passages; that is, the maximum value of the 1st and 2nd passage, of the 3rd and 4th passage, and of the 5th and 6th passage. The histogram of the maximum amplification ratios for each passage couple is plotted in Fig. SI.6, which also shows the cumulative probability distribution (starting from the largest value).

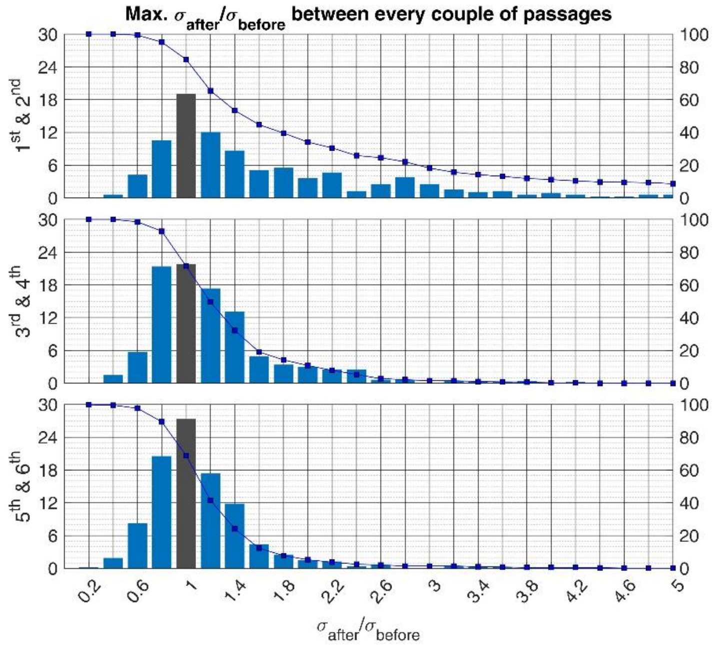

SI. 6: Probability distribution (in percentage, left scale of the panels) of the amplification ratio obtained for the different tide gauges. Each panel corresponds to a couple of odd-even passages (1st & 2nd, 3rd & 4th and 5th & 6th, from top to bottom). The maximum amplification ratio has been selected between the two passages of each pair. Each bar has a width of 0.2 in amplification ratio. The grey bar is centered at 1 and includes those tide gauges with amplification ratio from 0.9 to 1.1; it is outlined because the bars beyond that one correspond to tide gauges with an standard deviation increase greater than 10%.The cumulative probability distribution (i.e., the percentage of stations with amplifications ratios above a given value, right scale of the panels) has also been plotted (blue squares).

The Figure reveals that after the 1st or the 2nd passage more than 65% of the tide gauges showed an increase in the sea level signal standard deviation of at least a 10% (i.e. an amplification ratio greater than 1.1, this threshold is indicated in the figure with a dark grey bar) and almost 30% showed an amplification ratio greater than 2. The impact of the 3rd - 4th and of the 5th - 6th passages are much smaller, both in occurrence (between 50% and 40% of the stations have an amplification ratio greater than 1.1) and in magnitude (less than 10% have an amplification ratio greater than 2).
